# Harnessing the Anti-Tumor Mediators in Mast Cells as a New Strategy for Adoptive Cell Transfer for Cancer

**DOI:** 10.3389/fonc.2022.830199

**Published:** 2022-03-31

**Authors:** Mohammad Fereydouni, Mona Motaghed, Elnaz Ahani, Tal Kafri, Kristen Dellinger, Dean D. Metcalfe, Christopher L. Kepley

**Affiliations:** ^1^ Department of Nanoscience, Joint School of Nanoscience and Nanoengineering, University of North Carolina Greensboro (UNCG), Greensboro, NC, United States; ^2^ Department of Nanoengineering, Joint School of Nanoscience and Nanoengineering, North Carolina A&T State University, Greensboro, NC, United States; ^3^ Gene Therapy Center and Department of Microbiology and Immunology, University of North Carolina at Chapel Hill, Chapel Hill, NC, United States; ^4^ Laboratory of Allergic Diseases, National Institute of Allergy and Infectious Diseases, National Institutes of Health, Bethesda, MD, United States; ^5^ Department of Molecular and Cellular Sciences, Liberty University College of Osteopathic Medicine, Lynchburg, VA, United States

**Keywords:** mast cells, adoptive cell transfer, cancer immunotherapy, FcεRI, IgE

## Abstract

The emergence of cancer immunotherapies utilizing adoptive cell transfer (ACT) continues to be one of the most promising strategies for cancer treatment. Mast cells (MCs) which occur throughout vascularized tissues, are most commonly associated with Type I hypersensitivity, bind immunoglobin E (IgE) with high affinity, produce anti-cancer mediators such as tumor necrosis factor alpha (TNF-α) and granulocyte macrophage colony-stimulating factor (GM-CSF), and generally populate the tumor microenvironments. Yet, the role of MCs in cancer pathologies remains controversial with evidence for both anti-tumor and pro-tumor effects. Here, we review the studies examining the role of MCs in multiple forms of cancer, provide an alternative, MC-based hypothesis underlying the mechanism of therapeutic tumor IgE efficacy in clinical trials, and propose a novel strategy for using tumor-targeted, IgE-sensitized MCs as a platform for developing new cellular cancer immunotherapies. This autologous MC cancer immunotherapy could have several advantages over current cell-based cancer immunotherapies and provide new mechanistic strategies for cancer therapeutics alone or in combination with current approaches.

## Adoptive Cell Transfer for Cancer Immunotherapy

The use of autologous cells that can be targeted to tumors and induce apoptosis is an emerging therapeutic option to treat malignancies (1). From 2017 to 2018, there was a > 112% increase in the number of cell-based active agents in the global cancer immunotherapy pipeline. Most cells being investigated for autologous cancer immunotherapy have both pro- and anti-tumor mediators, their elevated numbers correlated with positive or negative patient outcomes, and strategies investigated to either inhibit their presence in tumors or utilize them for their anti-tumor properties. This strategy of adoptive cellular transfer (ACT) is typified by the use of autologous, peripheral T cells engineered *ex vivo* to express a transmembrane chimeric antigen receptor (CAR) composed of an extracellular, antigen-specific single-chain antibody and an intracellular T cell signaling domain (CAR T) (2). The use of CAR T-cell therapies has been approved by the Food and Drug Administration for children with acute lymphoblastic leukemia and adults with advanced lymphomas (3). Other T-cell based strategies, such as tumor-infiltrating lymphocyte (TIL) and engineered T cell receptor therapies are also being investigated (4). Several non-T immune cells also have potential anti-tumor activity. For example, dendritic cells (DC) modified *in vitro* with specific tumor-associated antigens to generate an immune response for cancer-cell elimination has led to clinical trials testing their safety and efficacy (5). Natural killer cells (NK) can eliminate cancer cells with surface markers associated with oncogenic transformation and have been investigated in clinical trials in patients with hematological malignancies or solid tumors (6). Peripheral blood eosinophils and neutrophils, containing potent mediators utilized by the immune system for pathogen destruction, have recently been demonstrated to have antitumorigenic activity (7, 8). As mentioned above, strategies to control tumor macrophages have resulted in numerous clinical trials in cancer patients to eliminate them alone or in combination with other therapies (9–11). Strategies to deplete macrophages are typified through inhibition of the CSF-1/CSF-1R signaling pathway. In general, depleting strategies have had limited success as unwanted removal of beneficial macrophages in non-tumor sights is a challenge (12). Conversely, other studies have hypothesized the anti-tumor capabilities of macrophages could be exploited and thus examined employing them as a form of ACT (13). While cytotoxic macrophages proved effective in animal models, this observation did not translate to humans (14). Recent strategies using CAR are intended to polarize pro-tumor and immunosuppressive M2 phenotype to a M1 phenotype with phagocytic functions, target cancer specific biomarkers, and induce an adaptive immune response (15, 16). In short, most cells being investigated as new platforms for cancer immunotherapy exert both pro- and anti-tumor effects. Therefore, the challenges moving forward in utilizing these cells is to remove the pro-tumor activity and/or enhance their anti-tumor functions. A summary table on the history of cell types being explored or used as cancer immunotherapy is shown in **Table 1**.

**Table 1:** Chronological history of cell-mediated cancer immunotherapy strategies.

Cell type	Year	Clinical trials	Strategy for targeting	Mechanism of action	Targeted cancer	Refs
**Bacteria cells**	1891	n/a	Injection of heat-killed cultures of bacteria into tumors to stimulate immune response.	Coley’s toxins released through the stimulation of TLRs on immune cells	Sarcoma, lymphoma, testicular carcinoma, etc.	(17)
**T cells**	1974	n/a	T cells exposed to histocompatible, virus-infected target cells lysed lymphocytic choriomeningitis-infected cells *in vitro* and *in vivo*.	T-cell activation and release of perforin and granzymes	Lymphocytic choriomeningitis	(18)
		(First cell-mediated cancer immunotherapy)				
**NK cells**	1975 to present	n/a	Endogenous type-C viruses in tumor led to immune cells reactivity in mice.	Tumor cell lysis with NK cells by secretion of IFN-γ, TNF-α, GM-CSF, and chemokines	YAC-1 lymphoma cell line	(19)
**Mycobacteria**	1990 and 1998	FDA approved	Attenuated live culture of bacteria injected in tumors to stimulate the innate immune response.	Macrophages phagocytosis	Non-muscle invasive bladder cancer	(20)
		ORR*=50%				
		PFS**=30m				
**Cytolytic T lymphocytes (CTLs)**	1991	n/a	Melanoma cells transduced with MZ2-E were recognized and killed by CTLs.	CTL activation and release of perforin and granzymes	Human melanoma	(21)
**T cell targeted immunomodulators**	1996-present	>60 FDA approved antibodies	Anti-PD-1/L1, anti-CTLA-4, Bispecific T-cell Engager (BiTE) antibodies, etc.	T-cell activation and release of perforin, granzymes, etc.	Colon carcinoma, fibrosarcoma, melanoma, bladder cancer	(22)
		ORR=12%-70%				
**Antigen presenting cells (APC)**	2010	FDA approved	GM-CSF/PAP fusion proteins induce APC activation and mobilized anti-PAP T cells.	Stimulation of T-cell immune response against PAP and release of perforin and granzymes	Prostate Cancer	(23)
		ORR= 32%				
		OS***				
**Dendritic cell (DC) vaccine**	1989-present	FDA approved	Immunization of mice with DC pulsed with unfractionated tumor proteins induced protective immunity against subsequent *in vivo* tumor cell challenge.	Antigen presentation by MHC I and CD8+ T cell secretion of perforin, granzymes, etc.	Malignant lymphomas stages III and IV, Breast cancers, etc.	(24)
**Dendritic cells**	2010-2020	Phase II completed	DC pulsed with melanoma specific peptides or tumor cell lysate stimulate response to melanoma cells.	Antigen presentation by MHC I and CD8+ T cell secretion of perforin, granzymes, etc.	Brain tumors	(25)
**CAR T cells**	2010-present	FDA approval 2017 and 2018.	T cells with chimeric antigen receptor to B cell CD19.	T-cell activation and release of perforin, granzymes, etc.	CD19+ B cell acute lymphoblastic leukemia	(26)
		ORR= 72%				
		PFS=9.2 m				
**Neutrophils**	2010-present	n/a	The anti-tumor activity of alemtuzumab was shown to be primarily dependent on the ADCC mediated by neutrophils *in vivo*.	G-CSF	B-cell lymphocytic leukemia	(8)
				GM-CSF		
**Macrophages**	2011-present	Used in several clinical trials as a combinatorial immunotherapy	Macrophages manipulated with antibodies or reprogrammed with metabolic/epigenetic substances to repolarize towards an anti-tumor phenotype	Downregulation of pro-tumor cytokines; Upregulation of anti-tumor cytokines	Pancreatic, melanoma, ovarian cancer, etc.	(27)
**Oncolytic viral particles**	2015	FDA approved	Viral particles modified to express GM-CSF for patients with melanoma	GM-CSF	Metastatic melanoma	(28)
		ORR=16%				
**Eosinophils**	2019	n/a	Adoptive transfer and cytokine neutralizations.	IL-5	Colorectal cancer	(7)
				INFγ		
**CAR Macrophages**	2020	n/a	Macrophages with chimeric antigen receptor to HER2/*neu* induced anti-tumor activity.	Phagocytosis,	HER2+ ovarian cancer, CD19+ leukemia	(15)
				MHC II,		
				TNF, INFγ		

*ORR: overall response rate.

**PFS: progression-free survival.

***OS: overall survival. n/a, not applicable.

## Challenges With Cell-Based Cancer Immunotherapies

While the numbers of autologous cells to target and inhibit cancer cell growth *in vivo* continues, so do the unanticipated roadblocks and challenges emerge. One challenge associated with CAR T cell therapies is the potentially life-threatening side-effect loosely defined as cytokine release syndrome (CRS). The CRS is induced by a systemic release of inflammatory cytokines associated with the T cell infusion and proliferation (and other T cell stimulants) (29). Also, the overwhelming majority of unique tumor antigens reside inside tumors, out of the reach of cells targeting them. This has led to efforts to identify and optimize delivery methods such as “*in situ* vaccination” at the tumor site hypothesized to release the inner tumor-associated antigens (30–33). Relatedly, most tumor antigens are promiscuous being found in and on cancerous and non-cancerous cells. This off-target phenomenon can result in serious or even fatal outcomes. An example of this is relates to an early trial in which T-cells were targeted to melanoma-associated antigen 3 (MAGE-A3) on metastatic cancers. Nervous system cells also express a similar MAGE-A12. As a result, T cells also invaded patients brain tissue resulting in the death of 2 out of 9 patients (34). The CAR T cell target CD19 is found on normal and malignant B cells. This can lead to lower immune cell numbers and side effects, such as a higher risk of infection when healthy cells are destroyed (35). Cancer cells are readily accessible to immune cells in blood as they circulate as individual cells or small clumps of cells compared to much larger solid tumors. Thus, another consideration in ACT development is the ability of the targeted cells to enter inside the tumor and release their anti-tumor mediators and killing at multiple locations.

## Allergy, Cancer Risk, and the Emergence of Tumor Targeting IgE’s for Cancer Immunotherapy

Epidemiological studies investigating a correlation between atopic disease (e.g. serum IgE levels) and several types of cancer have demonstrated either a protective role or as a risk factor depending on the location (36–38). The retrospective, epidemiological observations that dominate the literature in general evaluated self-reported allergy histories, total IgE measurements, and/or skin prick tests and risks of cancer. An emerging area of research that suggests that patients with “ultralow” IgE serum levels have an associated with high rates of new malignancies not observed in mice (39–41). Specifically, patients with IgE deficiency and negative skin prick tests had a higher rate of malignancy than patients who had IgE deficiency with positive skin tests (41). This is important as a hallmark of IgE mediated functional responses of tissue mast cells (MCs) is the skin prick test which would support the possibility that IgE bound to MCs may have a role in tumor surveillance. As the epidemiological evidence linking atopic status to cancer risk continues to evolve (increased, decreased, or no association) so have the proposed hypotheses attempting to relate the possible mechanism linking allergy to cancer (37).

The development of atopy is initiated by the generation of IgE which binds FcεRI on MCs and basophils to induce allergic mediator release which induces allergy inflammation when encountering allergen. Tumor targeting IgE’s are being developed in an attempt to harness the diverse acquired responses mediated through IgE (e.g. parasite expulsion), the success of targeting cancer tumor markers with humanized IgG as a therapeutic strategy (42), and the epidemiological evidence suggesting a protective role for atopy against some malignancies (43). The IgE isotype has several potential advantages over IgG antibodies approved by the FDA on the market to treat various cancers such as the low serum levels of IgE (generally 100,000 fold lower than IgG) that result in less competition for FcεR occupancy, lack of inhibitory FcεR, and induces a different anti-tumor immune response compared to IgG (44, 45). Currently, there are over 10 IgE antibodies derived from patients or produced to target tumor-specific that have been assessed using *in vitro* and *in vivo* cancer models (**Tables 2** and **3**) For example, Fu et al. investigated the serum levels of IgE in patients with pancreatic cancer and revealed the cytotoxic effect of the purified IgE against this type of cancer cells (49). The synthesized human tumor-specific IgE’s such as MOv18 IgE for ovarian carcinoma (47), Trastuzumab and C6MH3-B1 IgE’s for breast (50), colon (58), and ovarian (47) cancers, Cetuximab IgE for breast and epidermoid carcinoma (52), anti-hCD20 for human B-cell lymphoma (53), anti-PSA for human prostate cancer (55), have been investigated by many research groups (**Tables 2** and **3**). Of note, the MOv18 IgE specific for the folate receptor alpha (FRα) was demonstrated to have anti-tumor effects *in vitro* and *in vivo* and is in phase 1 clinical trials testing with early data demonstrating demonstrated safety and efficacy in ovarian cancer patients (64). The survival of FRα-positive xenograft-bearing mice was increased in the presence of monocytes (48). Systemic treatment with MOv18 IgE induced TNF-α and IL-10 upregulation in tumors and significantly upregulated TNF-α, MCP-1 and IL-10 levels in bronchoalveolar lavage fluid using an *in vivo* xenograft model (65). Further *in vitro* studies examined the anti-tumor mechanism of IgE and demonstrated pro-inflammatory signals and tumor cell killing by human monocytes (66). An IgE targeting the tumor-associated antigen SLC3A2 induced FcεRI-mediated degranulation using a rodent cell line transfected with human receptor and triggered with SLC3A2-positive cell lines (58). The antibody did not trigger human basophil activation using unfractionated peripheral blood from cancer patients. In each of these studies, the mechanistic emphasis was on IgE-monocyte-mediated anti-tumor effects *via* IgE Fc-mediated ADCC.

**Table 2:** *In-vitro* studies of IgE dependent cancer immunotherapy.

Year	Recombinant IgE	Name	Effector cells against cancer cells	Target cancer	Ref.
**1991**	Anti-HIV gp120	n/a	Human blood basophils and using IgE pathway for cancer immunotherapy	H2712 mouse mammary carcinoma	(46)
**1999**	Anti-FRα	MOv18 IgE	Human basophils and platelets against IGROV1 cell line	Ovarian carcinoma	(47)
**2003**	Anti-FRα	MOv18 IgE	Monocytes, eosinophils against human ovarian carcinoma cell line IGROV1	Human ovarian cancer	(48)
**2008**	IgE from patient	n/a	Peripheral blood mononuclear cells against HPAC cell line	Human pancreatic cancer	(49)
**2009**	Anti-HER2/*neu*	Trastuzumab IgE	Monocytic cell line U937 against SKBR3; Rat basophilic leukemia MC (RBL-SX38) expressing human FcεRI, against murine colon adenocarcinoma cell line CT26-HER2/*neu*	Human HER2/*neu* positive breast and colon cancers	(50)
**2011**	anti-FRα	MOv18 IgE	RBL SX-38 against ovarian carcinoma IGROV-1 cell line	Ovarian carcinoma	(51)
**2012**	Anti-EGFR	Cetuximab IgE	Purified human monocytes and MC, U937 and RBL-SX38 cell lines against EGFR epidermoid and breast cancer cell lines	Human breast cancer and epidermoid carcinoma	(52)
**2012**	Anti-hCD20	n/a	Primary human MC and eosinophils derived from umbilical cord blood against VU-3C6 hybridoma and OCI-Ly8 lymphoma cancer cell lines	Human B-cell non-Hodgkin lymphoma	(53)
**2012**	Anti-HER2/*neu*	C6MH3-B1	MC of transgenic mice strains that express human FcεRI against murine mammary carcinoma cells that express human HER2/*neu* (D2F2/E2)	Breast and ovarian cancer	(54)
**2013**	Anti-PSA	AR47.47 IgE	RBL-SX-38 cells sensitized with anti-PSA IgE and challenged with PSA or artificial molecules containing multiple epitopes of PSA	Human prostate cancer	(55)
**2017**	Anti-FRα	rMOv18 IgE/IgG2b	RBL-2H3 targeting WAG adenocarcinoma and ovarian tumor	FRα+ cancers	(56)
**2019**	Anti-HER2/*neu*	Trastuzumab IgE/C6MH3-B1 IgE	Human primary skin/adipose derived MC against breast cancer cell lines	Breast cancer	(57)
**2021**	SF-25	SLC3A2	RBL-SX-38 cell, basophils, cancer cell lines and *in vivo* xenograft models	Colon cancer (others)	(58)

n/a, not applicable.

**Table 3:** *In-vivo* studies of MC/IgE dependent cancer immunotherapy.

Year	IgE	Name	Animal	Anti-tumor mechanism/details	Target cancer	Ref.
**1999**	Anti-hFRα	MOv18 IgE	Mouse	Human peripheral blood mononuclear cells (PBMC) against IGROV1	Human ovarian carcinoma	(47)
**2012**	Anti-hHER2/*neu*	C6MH3-B1	Mouse	Mast cells of transgenic mice that express functional human FcεRI against D2F2/E2	Human breast and ovarian cancer	(54)
**2012**	Chimeric mouse-human anti-hMUCI	n/a	Chimeric mouse-human	Administration of anti-hMUC1 IgE significantly reduced growth of MUC1+ tumors in hFcεRI transgenic mice	Human breast carcinoma	(53)
**2013**	Anti-hPSA	AR47.47 IgE	Mouse	Mice immunized with PSA alone or in combination with anti-PSA IgE demonstrated effector cells’ activation but not systemic anaphylaxis	Human prostate cancer	(55)
**2014**	Anti-hFRα	MOv18 IgE	Cynomolgus monkey	Human and monkey PBMC against human U937 and IGROV1 cell line	Human ovarian carcinoma	(59)
**2015**	Anti-hFRα	MOv18 IgE	Human	In clinical trials phase I since 2015	Human ovarian cancer	(60)
**2016**	Anti-hHER2/*neu*	Trastuzumab/cetuximab IgG	Dog	HER-2 mimotope vaccines used in canine to assess safety and efficacy	Human HER2 positive breast cancer	(61)
**2017**	n/a	n/a	Mouse	Mice lacking multiple MC proteases (e.g. tryptase) exhibited higher extent of melanoma colonization compared to wild type animals	Mouse melanoma	(62)
**2017**	Anti-hFRα	hMOv18 IgE/IgG2b	Immunocompetent rat	Anti-folate receptor-α IgE, but not IgG recruits macrophages to attack tumors *via* TNF-α/MCP-1 signaling	Human FRα+ cancers such as ovarian	(56)
**2019**	Rat anti-hCSPG4 IgE	n/a	Rat	Immunocompetent mice bearing CSPG4+ tumor received systemic doses of IgE	Human melanoma, glioblastoma, and breast carcinoma	(63)
**2021**	SF-25	SLC3A2	Mouse	SLC3A2-specific IgE demonstrated cytotoxicity against tumor cells and longer overall survival	Colon cancer	(58)

n/a, not applicable.

## MC in Cancer; Evidence for Both Anti- and Pro-Tumor Roles

**Figure 1:**
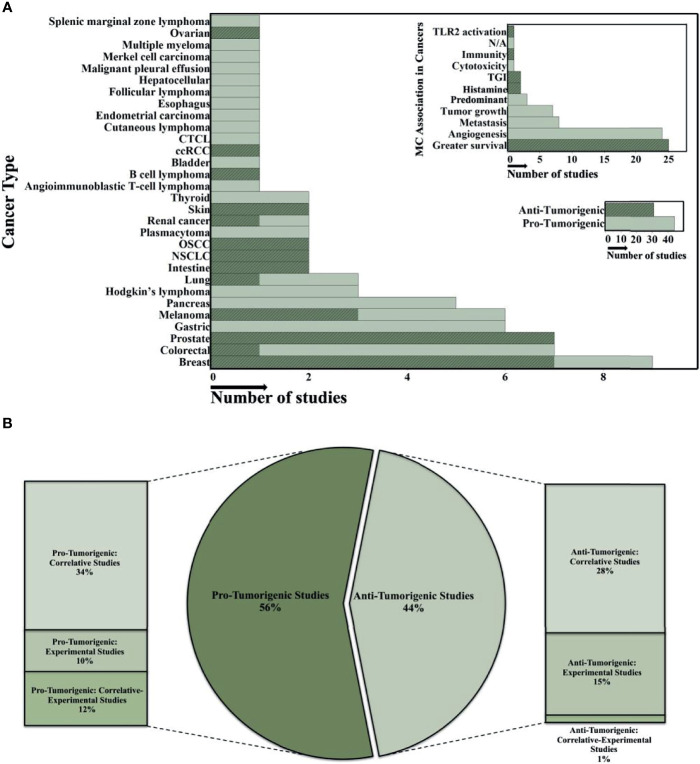
Overview of the role of human MC in different cancerous microenvironments. **(A)** The histograms summarize the data analysis from 75 published studies on MC's anti- or protumorigenic role in the various human cancer microenvironments. The y-axis shows cancer types and MC association in different tumor environments in the large and small histogram-top, respectively. The x-axis indicates the number of studies (all histograms). Highlighted regions demonstrate the number of anti-tumorigenic studies. JMP software was used to show the distribution of number of studies and finding across the categorical variables such as cancer type and MC association in tumor microenvironments in the 75 published studies. **(B)** The Bar-Pie chart illustrates the percentage of the 75 published studies which focused on either anti- or protumorigenic effects of MCs in various cancer microenvironments. In all studies, descriptive analysis is the primary evaluation strategy for MCs role in different cancer microenvironments. In the second step, most of the studies investigated either the Correlative, Experimental, or combination (Correlative-Experimental) approaches. Cutaneous T Cell Lymphomas (CTCL); clear-cell Renal Cell Carcinoma (ccRCC); Oral Squamous Cell Carcinoma (OSCC); Non-SmallCell Lung Cancer (NSCLC); Toll-Like Receptor 2 (TLR2); Tumor Growth Inhibitor (TGI). Predominant is predominancy of the numbers of infiltrated MCs that was investigated in some studies showing the pro-tumorigenic effect on some cancers at certain stages.

As mentioned above, MCs are the final tissue effector cell in FcεRI-IgE allergic responses through the release of histamine and other noxious mediators. Their ability to release these mediators is also controlled by non-IgE and non-receptor mechanisms that are less common and include hypoxia, adenosine, and certain chemokines within the tumor milieu (67). MCs possess both pro-tumor and anti-tumor mediators, are found in large numbers in and around many types of tumors, and studies have variously suggested MCs should be targets for inhibition/depletion or exploited as an anti-tumorigenic strategy (67). There are various studies that showed MCs have an anti-tumorigenic role in ovarian cancer (68), clear-cell renal cell carcinoma (ccRCC) (69), B cell lymphoma (70), skin cancer (71, 72), renal cancer (73), oral squamous cell carcinoma (OSCC) (74, 75), non-small-cell lung cancer (NSCLC) (76, 77), intestine cancer (71, 78), lung cancer (79), melanoma (80–82), prostate cancer (83–85), colorectal cancer (86), and breast cancer (57, 87–92) (**Figure 1A**). Patients with elevated MC counts had a significantly better event-free survival (EFS) compared to those with fewer MCs in several tumor types. Several unique phenotypic characteristics of MCs could contribute mechanistically to anti-tumor effects. Human MCs are unique in that they have prestored, releasable (through FcεRI) tumor necrosis factor alpha (TNF-α), histamine, and tryptase within their granules. The biggest impediment to using TNF-α as an anti-cancer agent is its systemic toxicity and strategies that limit its systemic distribution through local administration in patients have been investigated (93). Histamine induces the differentiation of immature myeloid cells and suppresses their ability to support the growth of tumor allografts (71). Increased histidine decarboxylase (which produces histamine) gene expression is associated with better relapse-free and overall survival in breast cancer patients and histamine treatment reduces tumor growth and increased apoptosis in xenograft breast cancer models (94). Mast cell tryptase alters the morphology and reduces the proliferation of human melanoma cells (82). We and others have demonstrated human MC release copious amounts (2,500-4,000 pg/ml from 10^5^ cells) of granulocyte-macrophage colony-stimulating factor (GM-CSF); also, an anti-tumor mediator investigated in over 50 clinical trials (95). Mast cells showed direct antitumor effects *in vitro* and decreased angiogenesis and recruitment of NK and T cells *in vivo (* 80).

In contrast, other studies have suggested a pro-tumorigenic role of MCs in different cancers (**Figure 1A**) with increased MC populations in certain tumor microenvironments associated with poor patient prognosis (96–106). These studies investigated the expression of MC markers (e.g. chymase/tryptase expression, FcεRI, c-KIT, etc.) in tumor tissues using immunohistochemistry, flow cytometry, immunoblotting, or RT-PCR techniques (67, 107, 108). In general, most published studies that attribute a pro-tumorigenic role for MC rely on correlations with increased MC numbers at a single time point, dependent on the tumor type, stage, and cancer microenvironment-and patient outcomes (**Figure 1B**). A “snapshot” analysis demonstrating an increase or decrease in MC numbers based on immunohistochemistry and subsequent association with a specific prognosis cannot be relied on to predict if these cells have a beneficial or deleterious effect. Observing an increase in MC numbers paralleled by a poor prognosis (or vice versa) demonstrates a correlation, not a causation between numbers and prognosis. Studies are needed to assess the effects selectively knocking down (i.e. CRISPR) the pro-tumorigenic and/or upregulation of anti-tumorigenic mediators from human MCs. Nonetheless, MCs are one of the first cells to infilitrate the tumor microenvironment and possess such a wide range of receptors and molecules with diverse functions that mediate tumor responses that adds to the controversial role they play in the disease (109).

Another issue surrounding the analysis of the MCs role in cancers relates to conclusions drawn from MC knockout studies, with constraints in results observed depending on the model (110–112). In some cases, a pro- and anti-tumor effect was observed in the same tumors (67, 113, 114). In addition, differences in MC phenotypic and functional responses between mice and humans have been well documented (111, 115–123). For example, Fcγ receptor expression and functional responses mediated by them on mouse and human MCs and monocytes are vastly different (124–126). Further, mouse MCs have a diverse range of various proteases (127) while human MCs principally express three proteases (tryptase, chymase, and carboxypeptidase-A) (128). Histamine is released from human MCs, while both serotonin and histamine are liberated in reasonable amounts from MCs in mice, and both contribute to the physiological effects in anaphylactic reactions, respectively in these species. Interleukin-3 has a profound effect on murine MC differentiation and function not observed with human MCs. Of course, cancer therapeutic strategies require animal models to determine efficacy of drug targets, safety, biodistribution, etc. But caution must be taken when extrapolating data from mouse models of cancer, especially when focused on MC numbers and MC Fc-specific mechanisms.

## Could MCs Mediate the Efficacy of Anti-Tumor IgE’s and in IgE Tumor Surveillance?

The mechanisms underlying the anti-tumor effects of therapeutic IgE’s are mostly attributed to monocyte and macrophage infiltration and subsequent IgE-mediated activation of these cells around tumors (56, 65, 129, 130). This hypothesized mechanism seems counter-intuitive to current evidence that demonstrates tumor-infiltrating myeloid cells promote, rather than inhibit-cancer progression (10). FcϵRIα-positive macrophages have been identified as critical infiltrating cells that induce tumor progression in squamous cell carcinoma (131) [although evidence is presented that the anti-FcϵRI antibody used in this study was not specific for FcϵRI on macrophages (132)]. As is the case with MC, macrophages may initiate, promote, or suppress the development of cancer, possess both pro (e.g. VEGF, EGF, and TGF-β) and inhibitory (e.g. nitric oxide), and have been implicated to mediate angiogenesis, invasiveness, metastasis, and acquired resistance to therapeutic strategies largely based on correlations between cell numbers and patient outcomes (133–135).

The hypothesis that monocytes/macrophages mediate anti-tumor efficacy to tumor IgE’s is also premised on the surface expression of FcεRI on monocytes/macrophages that controls their effector functions. However, the expression of FcεRI on primary human monocytes has been reported to be low (<10% in non-atopic patients), or not at all, compared to primary human MC and the expression level on monocytes is 10 to 100-fold less than observed for peripheral blood basophils from the same subjects (136, 137). While human monocytes can be manipulated to increase FcεRI expression *in vitro (* 66) it is unknown if primary, tissue macrophages express FcεRI to any degree in humans. It also cannot be assumed the expression of IgE receptors will stay the same after entry and maturation in the tissues as monocytes undergo phenotypic changes upon tissue entry as they mature into macrophages (138). Others have shown human tissue macrophages do not express FcεRI (139–141). Here, another difference between species relates to reports in rodent studies that support the conclusion that macrophages can mediate anaphylaxis in mice; a phenomenon not described in humans (142, 143). One study showed that the responses of human alveolar macrophages involving IgE *in vitro (* 144, 145) was most probably mediated by FcεRII (CD23) which has lower affinity for IgE, is distinct functionally from FcεRI (146, 147), and would help explain the RBC-rosetting most of these older studies used to determine IgE binding (148, 149). Lastly, other tissue cells besides MC have been reported to express FcεRI (e.g. Langerhan cells) and the low affinity receptor for IgE (150). Human basophils (and in some cases eosinophils) express FcεRI they are not normally found in tissue but are recruited following certain pathological mechanisms (151). Human eosinophils have demonstrated FcεRI expression (and have anti-tumor properties (7)) but only from donors with eosinophilia and lymphomas (152). Thus, the likelihood of tumor specific IgE binding to human monocyte-derived, tissue macrophages with unknown FcεRI expression to mediate effects seems less likely given many other IgE binding cells are present. MCs [with almost 100% FcεRI expression (57)] are as abundant or more abundant in the tumor microenvironment than macrophages depending on the tumor type. For example, the rodent form of IgE MOv18 reduced lung metastases in a syngeneic rat tumor model expressing human FRα which was attributed to TNFα, IL-10, and MCP-1 released by MOv18-triggered monocytes (56). However, the cytokine profile induced in BAL by MOv18 (TNFα, MCP-1, and IL-10) could very likely include a contribution from lung MCs which we and many others have shown produce such cytokines upon FcεRI stimulation (57, 153–156). We thus propose the binding of tumor targeted IgE Fc to human MC FcεRI and subsequent triggering of this receptor upon tumor engagement mediate the anti-tumor effects of therapeutic IgE’s given the demonstrated high amounts of FcεRI on primary human MCs in the tumor milieu (157), the high numbers of FcεRI (>1 x 10^5^/cell) that require only ≈100 receptors for full activation (67, 158), the affinity of this interaction (159), the juxtaposition of MCs with cancers cells (67), and the anti-tumor mediators released from MCs (160). Infusion of IgE into patients is hypothesized to increase surface expression of MC FcεRI as this receptor is dependent on serum IgE levels (158, 161).

## Studies Using Tumor Targeting IgE’s and MCs

Attempts to utilize anti-tumor mediators from MCs for cancer cell targeting was first examined using a mouse–human chimeric IgE specific for CD20 and the epithelial antigen MUC1. Cord blood-derived MCs sensitized with anti-hCD20 IgE are cytotoxic to CD20 tumor cells *in vitro (* 53). We used adipose-derived mast cells (ADMC) sensitized with human anti-HER2/*neu* IgE which bound to and released MC mediators when incubated with HER2/*neu*-positive human breast cancer cells (SK-BR-3 and BT-474) resulting in TNF-α mediated, tumor cell apoptosis (57). Importantly, monomeric (shed) HER2/*neu* and serums from HER2+ breast cancer patients did not induce ADMC degranulation, suggesting that such an interaction will not trigger systemic anaphylaxis.

## Will MC be Added to Growing List of Tumor Targeting Cellular Immunotherapy?

As discussed above the variety of cell types being investigated as new strategies for cancer immunotherapy continues to increase. MCs are similar to tumor associated macrophages as discussed above in that both have both pro- and anti-tumor capabilities and correlative studies led to assumptions regarding their role in various cancers (16). Because of this, initial efforts were aimed at depleting or repolarizing these cells as a therapeutic, anti-tumor strategy. MCs are presently at the apparently contradictory position in which rationale arguments could be made for inhibiting their numbers in the tumor milieu or increasing their numbers and harnessing their natural associated anti-tumor mediators within them. Yet from our perspective informed decisions as to deplete, increase, or repolarize MCs cannot be made until more studies assess their functional role in cancer models. As with human macrophages, human MC may need to be “repolarized” from a Type I hypersensitivity-associated cell type to an anti-cancer cell through up or down regulation of certain mediators. To this end, transfection/transduction of primary MCs has only recently been achieved using human peripheral blood derived MCs (162). The conditions that will now allow us to manipulate MC so that maximal anti-tumor activity is conferred and/or potential deleterious mediators can be deleted are being explored in our laboratory.

**Figure 2:**
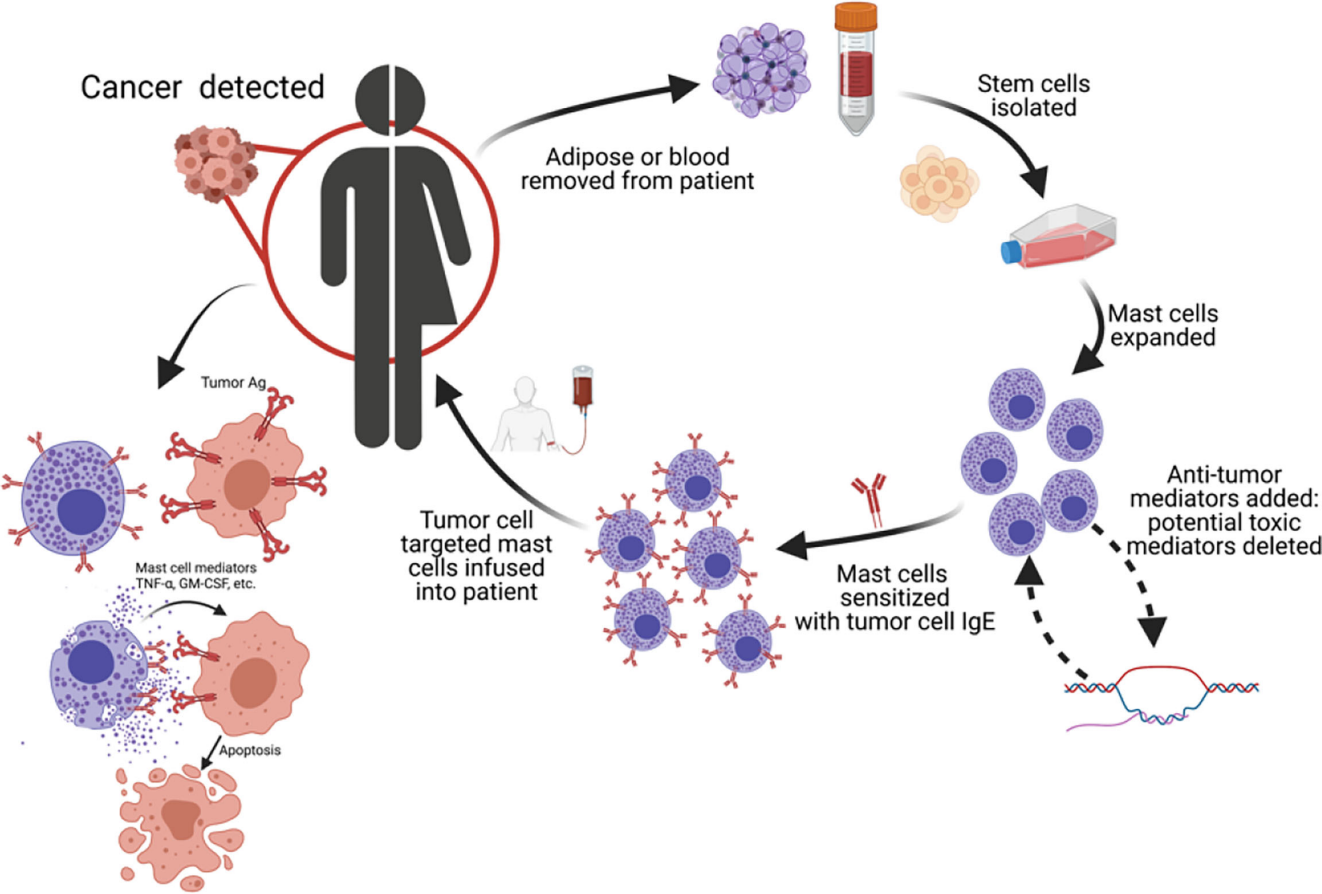
Autologous MC cancer immunotherapy; a potential new platform for cancer therapy. We propose using MCs as a new cell type for adoptive cell transfer for cancers in which tumor-specific IgE’s are available. MC progenitors are obtained from patient, MCs expanded and polarized to enhance cytotoxicity and/or minimize systemic toxicity, and re-polarized MCs reinfused into patient.

We propose human MCs as another cell type to be used in ACT for cancers in which tumor specific IgE’s are available or could be made. To do this, autologous MCs could be obtained from adipose tissue or cultured from peripheral blood and expanded *ex vivo*. Anti-tumor capabilities could be increased or deleterious mediators downregulated during expansion. FcεRI-positive MCs are then sensitized with IgE targeting antigens found on tumors. The tumor targeting MCs would then injected into the patient and become active upon FcεRI-IgE crosslinking. This autologous MC cancer immunotherapy (**Figure 2**) would result in the release of anti-tumor mediators within the tumor milieu (see graphical abstract). Recently we have demonstrated up to 6 x 10^6^ human ADMC can be injected i.v. into mice with no toxicological effects. The ADMC, sensitized with human IgE recognizing the breast cancer antigen HER2/*neu*, shrink HER2/*neu*-positive tumors *in vivo* using a xenograft mouse model (manuscript submitted). Since human GM-CSF is not active in mice (163) the anti-tumor effects we have observed are expected to be stronger in humans in which GM-CSF would be fully active (164). This approach may enhance anti-tumor immunity through epitope spreading of cancer antigens. Importantly, this strategy may spurn new areas of research through transformation or manufacturing of tumor-targeted IgE’s. Harvesting adipose tissue from patients is not difficult, commonly performed, and increasingly being used for a wide variety of clinical applications (165, 166). Recently, we have demonstrated peripheral blood, CD34-positive stem cell derived MC also have anti-tumor activity providing a second source of autologous MCs (data not shown).

## Advantages of AMCIT as a New Cancer Immunotherapy Approach

There is a growing list of human IgE antibodies targeting cancer antigens that have been fully characterized which provide the targeting needed to transport the MCs to the tumor sites (43). It should be noted with caution when examining anti-tumor effects experimentally that the use of certain tumor targeted IgE’s is limited as human IgE does not bind mouse FcεRI receptors (167). Second, the *in vitro* incubation of MCs with IgE for targeting is extremely stable (168) and allows for the saturation of FcεRI binding, thus maximizing the effect while preventing patient IgE binding. IgE also stays bound to MCs for several months *in vivo (* 169–173). Third, the adipose stem cells may be cryopreserved, reconstituted, and differentiated into ADMC while retaining expression of introduced genetic modules (data not shown). This is an important characteristic, as it greatly enhances the “logistics” of the potential therapy in that patient cells could be transduced, cryopreserved for shipping, and reconstituted when needed for therapy. Fourth, MC activation is hypothesized to induce acute inflammation and destruction of cancer cells in the tumor microenvironment due to the release of multiple mediators. The presence of dead tumor cells would allow uptake and presentation of tumor antigens by antigen presenting cells as with dendritic cells that elicit an adaptive, long lasting immune response not only to the targeted antigen but also to other tumor antigens due to epitope spreading. This would increase due to the local release of GM-CSF from MCs (174, 175) and the release of regulatory T-cell function suppressors (176). Of course, the use of tumor IgE’s alone or using tumor IgE-sensitized MCs as proposed here has the obvious potential to induce a systemic allergic response. Strategies to delete select mediators in human MCs are underway but with caution as it is simply not known if those with potential “toxic” side effects also have potent anti-tumor effects. Lastly, this strategy has the potential to circumvent challenges associated with current ACT strategies in which hyperactive T cells create a cytokine storm (177), reduced chances of off-target binding (not expressed on normal cells; e.g. CD19) (178), and avoid the lack of expansion and/or persistence of autologous cells (as with NK cells) (179).

## Conclusions and Future Directions

While the role of MCs in all cancer pathogenesis is still unclear, future studies are needed to examine if *ex vivo*-derived MCs possess anti-tumor capabilities. Questions remain regarding the possibility of systemic MC activation, although this issue can be overcome as discussed above with prophylactic anti-histamines, as is common practice (180–183). An alternative clinical strategy could be to first add IgE, followed by the MCs. Antigen levels on target cells may vary in patients, which could minimize cell targeting and activation. It is not anticipated that high levels of antigen will be needed, as human MCs require ≈100 FcεRI receptors to aggregate for a full activation response (158) and all FcεRI will be saturated so that patient IgE binding will not occur. Shed antigen in serum may also “mask” the MC-bound IgE without inducing degranulation, however blocking future binding. That said, this remains unlikely given the *in vivo* studies using IgE antibodies to tumor antigens do not suggest masking (50, 58, 184, 185).

There are myriad reasons to speculate on the many potential roadblocks that could arise during the development of the AMCIT as a new cancer immunotherapy strategy. But it is important to highlight similar misgivings, inaccurate predictions regarding toxicity, and major setbacks in the early years of CAR T-cell therapy (177, 186, 187). The emergence of CAR-T immunotherapy was met with skepticism and progressed only gradually based on incremental insights over many years. Even though unexpected toxic effects in Phase 1 studies can quell any new therapy, the unfortunate reality is that it can take time to distinguish toxic effects as was the case in the first CART-19 trials (186, 188, 189). The point is that it is impossible to predict what, if any, side effects might occur *in vivo* with ADMC until studies to assess their role are performed. We believe the need for novel therapies that bring new mechanisms to combat cancer pathologies are important to investigate given the continued morbidity and mortality associated with this disease.

## Author Contributions

Conceptualization: CK. Methodology: MF, MM, EA, KD, TK, and CK. Investigation: MF, MM, EA, KD, TK, DM, and CK. Writing-original draft: MF and CK. Writing-review and editing: MM, MF, EA, KD, TK, DM, and CK. Funding acquisition: CK and TK. Resources: DM, TK, and CK. Supervision: DM, TK, and CK. All authors contributed to the article and approved the submitted version.

## Funding

CK was funded by NIH/NCI grant number 1R15CA246430, Specialized Center of Research grant and Pilot grant from UNC-Chapel Hill, Lineberger Cancer Center, and Giant Steps grant from UNCG. DM is supported by the Division of Intramural Research, NIH/NIAID. TK is funded by NIH R01HL155986.

## Conflict of Interest

The authors declare that the research was conducted in the absence of any commercial or financial relationships that could be construed as a potential conflict of interest.

## Publisher’s Note

All claims expressed in this article are solely those of the authors and do not necessarily represent those of their affiliated organizations, or those of the publisher, the editors and the reviewers. Any product that may be evaluated in this article, or claim that may be made by its manufacturer, is not guaranteed or endorsed by the publisher.

## Abbreviations

MC, Mast Cell; MCs, Mast Cells; ADMC, Adipose-Derived Mast Cells; IgE, Immunoglobin E; TNF-α, Tumor Necrosis Factor Alpha; GM-CSF, Granulocyte-Macrophage Colony-Stimulating Factor; AMCIT, Autologous MC Cancer Immunotherapy; ACT, Adoptive Cell Transfer; CAR, Chimeric Antigen Receptor; CAR T, Chimeric Antigen Receptor T Cells; TIL, Tumor-Infiltrating Lymphocyte; CRS, Cytokine Release Storm; EFS, Event-Free Survival; ORR, Overall Response Rate; PFS, Progression-Free Survival; OS, Overall Survival; ADCC, Antibody-Dependent Cellular Cytotoxicity; FDA, US Food and Drug Administration; NK, Natural Killer Cells; DC, Dendritic Cells.

## References

[1] Emens LA Ascierto PA Darcy PK Demaria S Eggermont AMM Redmond WL. Cancer Immunotherapy: Opportunities and Challenges in the Rapidly Evolving Clinical Landscape. Eur J Cancer (2017) 81:116–29. doi: 10.1016/j.ejca.2017.01.035 28623775

[2] June CH O’Connor RS Kawalekar OU Ghassemi S Milone MC. CAR T Cell Immunotherapy for Human Cancer. Science (2018) 359(6382):1361–5. doi: 10.1126/science.aar6711 29567707

[3] Tran E Longo DL Urba WJ. A Milestone for CAR T Cells. N Engl J Med (2017) 377(26):2593–6. doi: 10.1056/NEJMe1714680 29226781

[4] Greenberg PD. How Cellular Immunotherapies Are Changing the Outlook for Cancer Patients. Institute CR. In: editor. Cancer Research Institute: CRI Documents (2022).

[5] Huber A Dammeijer F Aerts J Vroman H. Current State of Dendritic Cell-Based Immunotherapy: Opportunities for *In Vitro* Antigen Loading of Different DC Subsets? Front Immunol (2018) 9:2804. doi: 10.3389/fimmu.2018.02804 30559743 PMC6287551

[6] Shimasaki N Jain A Campana D. NK Cells for Cancer Immunotherapy. Nat Rev Drug Discov (2020) 19(3):200–18. doi: 10.1038/s41573-019-0052-1 31907401

[7] Reichman H Itan M Rozenberg P Yarmolovski T Brazowski E Varol C. Activated Eosinophils Exert Antitumorigenic Activities in Colorectal Cancer. Cancer Immunol Res (2019) 7(3):388–400. doi: 10.1158/2326-6066.CIR-18-0494 30665890

[8] Furumaya C Martinez-Sanz P Bouti P Kuijpers TW Matlung HL. Plasticity in Pro- and Anti-Tumor Activity of Neutrophils: Shifting the Balance. Front Immunol (2020) 11:2100. doi: 10.3389/fimmu.2020.02100 32983165 PMC7492657

[9] Wu K Lin K Li X Yuan X Xu P Ni P. Redefining Tumor-Associated Macrophage Subpopulations and Functions in the Tumor Microenvironment. Front Immunol (2020) 11:1731. doi: 10.3389/fimmu.2020.01731 32849616 PMC7417513

[10] Allavena P Anfray C Ummarino A Andon FT. Therapeutic Manipulation of Tumor-Associated Macrophages: Facts and Hopes From a Clinical and Translational Perspective. Clin Cancer Res (2021) 27(12):3291–7. doi: 10.1158/1078-0432.CCR-20-1679 33531428

[11] Cheng N Bai X Shu Y Ahmad O Shen P. Targeting Tumor-Associated Macrophages as an Antitumor Strategy. Biochem Pharmacol (2021) 183:114354. doi: 10.1016/j.bcp.2020.114354 33279498

[12] Poh AR Ernst M. Targeting Macrophages in Cancer: From Bench to Bedside. Front Oncol (2018) 8:49. doi: 10.3389/fonc.2018.00049 29594035 PMC5858529

[13] Anderson NR Minutolo NG Gill S Klichinsky M. Macrophage-Based Approaches for Cancer Immunotherapy. Cancer Res (2021) 81(5):1201–8. doi: 10.1158/0008-5472.CAN-20-2990 33203697

[14] Andreesen R Hennemann B Krause SW. Adoptive Immunotherapy of Cancer Using Monocyte-Derived Macrophages: Rationale, Current Status, and Perspectives. J Leuk Biol (1998) 64(4):419–26. doi: 10.1002/jlb.64.4.419 9766621

[15] Klichinsky M Ruella M Shestova O Lu XM Best A Zeeman M. Human Chimeric Antigen Receptor Macrophages for Cancer Immunotherapy. Nat Biotechnol (2020) 38(8):947–53. doi: 10.1038/s41587-020-0462-y PMC7883632 32361713

[16] Mukhopadhyay M. Macrophages Enter CAR Immunotherapy. Nat Methods (2020) 17(6):561. doi: 10.1038/s41592-020-0862-4 32499619

[17] Coley WB. The Classic: The Treatment of Malignant Tumors by Repeated Inoculations of ErysipelasWith a Report of Ten Original Cases. Clin Orthop Relat Res (1991) 262:3–11. doi: 10.1097/00003086-199101000-00002 1984929

[18] Zinkernagel RM Doherty PC. Immunological Surveillance Against Altered Self Components by Sensitised T Lymphocytes in Lymphocytes Choriomeningitis. Nature (1974) 251(5475):547–8. doi: 10.1038/251547a0 4547543

[19] Herberman RB Nunn ME Holden HT Lavrin DH. Natural Cytotoxic Reactivity of Mouse Lymphoid Cells Against Syngeneic and Allogeneic Tumors. II. Characterization of Effector Cells. Int J Cancer (1975) 16(2):230–9. doi: 10.1002/ijc.2910160205 1080480

[20] Organon Teknika Corporation. Summary for Basis of Approval (1998). Available at: http://waybackarchive-itorg/7993/20170723144330/https://wwwfdagov/downloads/BiologicsBloodVaccines/Vaccines/ApprovedProducts/UCM101490pdf (Accessed November 1, 2021).

[21] van der Bruggen P Traversari C Chomez P Lurquin C De Plaen E Van den Eynde B. A Gene Encoding an Antigen Recognized by Cytolytic T Lymphocytes on a Human Melanoma. Science (1991) 254(5038):1643–7. doi: 10.1126/science.1840703 1840703

[22] Cancer Research Institute. FDA Approval Timeline of Active Immunotherapies. Available at: https://wwwcancerresearchorg/scientists/immuno-oncology-landscape/fda-approval-timeline-of-active-immunotherapies (Accessed August 5, 2021).

[23] Gardner TA Elzey BD Hahn NM. Sipuleucel-T (Provenge) Autologous Vaccine Approved for Treatment of Men With Asymptomatic or Minimally Symptomatic Castrate-Resistant Metastatic Prostate Cancer. Hum Vaccin Immunother (2012) 8(4):534–9. doi: 10.4161/hv.19795 22832254

[24] Fecci PE Mitchell DA Archer GE Morse MA Lyerly HK Bigner DD. The History, Evolution, and Clinical Use of Dendritic Cell-Based Immunization Strategies in the Therapy of Brain Tumors. J Neurooncol (2003) 64(1-2):161–76. doi: 10.1007/BF02700031 12952297

[25] Clinincal trials phase II. Dendritic Cell Vaccine for Patients With Brain Tumors. Available at: https://clinicaltrialsgov/ct2/show/NCT01204684 (Accessed June 1, 2021).

[26] Mohanty R Chowdhury CR Arega S Sen P Ganguly P Ganguly N. CAR T Cell Therapy: A New Era for Cancer Treatment (Review). Oncol Rep (2019) 42(6):2183–95. doi: 10.3892/or.2019.7335 31578576

[27] DeNardo DG Ruffell B. Macrophages as Regulators of Tumour Immunity and Immunotherapy. Nat Rev Immunol (2019) 19(6):369–82. doi: 10.1038/s41577-019-0127-6 PMC7339861 30718830

[28] U.S. Food and Drug Administration. IMLYGIC (Talimogene Laherparepvec, Amgen Inc.). Available at: https://wwwfdagov/vaccines-blood-biologics/cellular-gene-therapy-products/imlygic-talimogene-laherparepvec (Accessed November 21, 2021).

[29] Hirayama AV Turtle CJ. Toxicities of CD19 CAR-T Cell Immunotherapy. Am J Hematol (2019) 94(S1):S42–s49. doi: 10.1002/ajh.25445 30784102

[30] Hammerich L Bhardwaj N Kohrt HE Brody JD. *In Situ* Vaccination for the Treatment of Cancer. Immunotherapy (2016) 8(3):315–30. doi: 10.2217/imt.15.120 26860335

[31] Han S Wang W-W Rozali E Toh HC. Defining Precision Cellular Immunotherapy—Seeking Biomarkers to Predict and Optimize Outcomes of T Cell Therapies in Cancer. Precis Cancer Med (2019) 2:25. doi: 10.21037/pcm.2019.07.02

[32] Riley RS June CH Langer R Mitchell MJ. Delivery Technologies for Cancer Immunotherapy. Nat Rev Drug Discov (2019) 18(3):175–96. doi: 10.1038/s41573-018-0006-z PMC6410566 30622344

[33] Zhang H Ye ZL Yuan ZG Luo ZQ Jin HJ Qian QJ. New Strategies for the Treatment of Solid Tumors With CAR-T Cells. Int J Biol Sci (2016) 12(6):718–29. doi: 10.7150/ijbs.14405 PMC4870715 27194949

[34] Morgan RA Chinnasamy N Abate-Daga D Gros A Robbins PF Zheng Z. Cancer Regression and Neurological Toxicity Following Anti-MAGE-A3 TCR Gene Therapy. J Immunother (2013) 36(2):133–51. doi: 10.1097/CJI.0b013e3182829903 PMC3581823 23377668

[35] American Cancer Society. CAR T-Cell Therapy and Its Side Effects. Available at: https://wwwcancerorg/treatment/treatments-and-side-effects/treatment-types/immunotherapy/car-t-cell1html (Accessed June 10, 2021).

[36] Cui Y Hill AW. Atopy and Specific Cancer Sites: A Review of Epidemiological Studies. Clin Rev Allergy Immunol (2016) 51(3):338–52. doi: 10.1007/s12016-016-8559-2 27277132

[37] Kozłowska R Bożek A Jarząb J. Association Between Cancer and Allergies. Allergy Asthma Clin Immunol (2016) 12:39–9. doi: 10.1186/s13223-016-0147-8 PMC4982132 27525013

[38] Bozek A Jarzab J Mielnik M Bogacz A Kozlowska R Mangold D. Can Atopy Have a Protective Effect Against Cancer? PLoS One (2020) 15(2):e0226950. doi: 10.1371/journal.pone.0226950 32015564 PMC6996965

[39] Ferastraoaru D Jordakieva G Jensen-Jarolim E. The Other Side of the Coin: IgE Deficiency, A Susceptibility Factor for Malignancy Occurrence. World Allergy Organ J (2021) 14(1):100505. doi: 10.1016/j.waojou.2020.100505 33664932 PMC7887422

[40] Ferastraoaru D Bax HJ Bergmann C Capron M Castells M Dombrowicz D. AllergoOncology: Ultra-Low IgE, a Potential Novel Biomarker in Cancer-a Position Paper of the European Academy of Allergy and Clinical Immunology (EAACI). Clin Transl Allergy (2020) 10(32):020–00335. doi: 10.1186/s13601-020-00335-w PMC7366896 32695309

[41] Ferastraoaru D Rosenstreich D. IgE Deficiency Is Associated With High Rates of New Malignancies: Results of a Longitudinal Cohort Study. J Allergy Clin Immunol Pract (2020) 8(1):413–5. doi: 10.1016/j.jaip.2019.06.031 31295561

[42] Goydel RS Rader C. Antibody-Based Cancer Therapy. Oncogene (2021) 4(10):021–01811. 10.1038/s41388-021-01811-8 PMC8357052 33947958

[43] Leoh LS Daniels-Wells TR Penichet ML. IgE Immunotherapy Against Cancer. Curr Top Microbiol Immunol (2015) 388:109–49. doi: 10.1007/978-3-319-13725-4_6 PMC4452505 25553797

[44] Clynes RA Towers TL Presta LG Ravetch JV. Inhibitory Fc Receptors Modulate *In Vivo* Cytoxicity Against Tumor Targets. Nat Med (2000) 6(4):443–6. doi: 10.1038/74704 10742152

[45] Nimmerjahn F Ravetch JV. Antibodies, Fc Receptors and Cancer. Curr Opin Immunol (2007) 19(2):239–45. doi: 10.1016/j.coi.2007.01.005 17291742

[46] Sun LK Liou RS Sun NC Gossett LA Sun C Davis FM. Transfectomas Expressing Both Secreted and Membrane-Bound Forms of Chimeric IgE With Anti-Viral Specificity. J Immunol (1991) 146(1):199–205. 1701791

[47] Gould HJ Mackay GA Karagiannis SN O’Toole CM Marsh PJ Daniel BE. Comparison of IgE and IgG Antibody-Dependent Cytotoxicity *In Vitro* and in a SCID Mouse Xenograft Model of Ovarian Carcinoma. Eur J Immunol (1999) 29(11):3527–37. doi: 10.1002/(SICI)1521-4141(199911)29:11<3527::AID-IMMU3527>3.0.CO;2-5 10556807

[48] Karagiannis SN Wang Q East N Burke F Riffard S Bracher MG. Activity of Human Monocytes in IgE Antibody-Dependent Surveillance and Killing of Ovarian Tumor Cells. Eur J Immunol (2003) 33(4):1030–40. doi: 10.1002/eji.200323185 12672069

[49] Fu SL Pierre J Smith-Norowitz TA Hagler M Bowne W Pincus MR. Immunoglobulin E Antibodies From Pancreatic Cancer Patients Mediate Antibody-Dependent Cell-Mediated Cytotoxicity Against Pancreatic Cancer Cells. Clin Exp Immunol (2008) 153(3):401–9. doi: 10.1111/j.1365-2249.2008.03726.x PMC2527370 18803764

[50] Karagiannis P Singer J Hunt J Gan SK Rudman SM Mechtcheriakova D. Characterisation of an Engineered Trastuzumab IgE Antibody and Effector Cell Mechanisms Targeting HER2/neu-Positive Tumour Cells. Cancer Immunol Immunother (2009) 58(6):915–30. doi: 10.1007/s00262-008-0607-1 PMC3017872 18941743

[51] Rudman SM Josephs DH Cambrook H Karagiannis P Gilbert AE Dodev T. Harnessing Engineered Antibodies of the IgE Class to Combat Malignancy: Initial Assessment of FcvarepsilonRI-Mediated Basophil Activation by a Tumour-Specific IgE Antibody to Evaluate the Risk of Type I Hypersensitivity. Clin Exp Allergy (2011) 41(10):1400–13. doi: 10.1111/j.1365-2222.2011.03770.x 21569129

[52] Spillner E Plum M Blank S Miehe M Singer J Braren I. Recombinant IgE Antibody Engineering to Target EGFR. Cancer Immunol Immunother (2012) 61(9):1565–73. doi: 10.1007/s00262-012-1287-4 PMC11028481 22674055

[53] Teo PZ Utz PJ Mollick JA. Using the Allergic Immune System to Target Cancer: Activity of IgE Antibodies Specific for Human CD20 and MUC1. Cancer Immunol Immunother (2012) 61(12):2295–309. doi: 10.1007/s00262-012-1299-0 PMC3833261 22692757

[54] Daniels TR Leuchter RK Quintero R Helguera G Rodriguez JA Martinez-Maza O. Targeting HER2/neu With a Fully Human IgE to Harness the Allergic Reaction Against Cancer Cells. Cancer Immunol Immunother (2012) 61(7):991–1003. doi: 10.1007/s00262-011-1150-z 22127364 PMC3719406

[55] Daniels-Wells TR Helguera G Leuchter RK Quintero R Kozman M Rodriguez JA. A Novel IgE Antibody Targeting the Prostate-Specific Antigen as a Potential Prostate Cancer Therapy. BMC Cancer (2013) 13:195. doi: 10.1186/1471-2407-13-195 23594731 PMC3651304

[56] Josephs DH Bax HJ Dodev T Georgouli M Nakamura M Pellizzari G. Anti-Folate Receptor-Alpha IgE But Not IgG Recruits Macrophages to Attack Tumors *via* TNFalpha/MCP-1 Signaling. Cancer Res (2017) 77(5):1127–41. doi: 10.1158/0008-5472.CAN-16-1829 PMC6173310 28096174

[57] Plotkin JD Elias MG Fereydouni M Daniels-Wells TR Dellinger AL Penichet ML. Human Mast Cells From Adipose Tissue Target and Induce Apoptosis of Breast Cancer Cells. Front Immunol (2019) 10:138. doi: 10.3389/fimmu.2019.00138 30833944 PMC6387946

[58] Pellizzari G Martinez O Crescioli S Page R Di Meo A Mele S. Immunotherapy Using IgE or CAR T Cells for Cancers Expressing the Tumor Antigen SLC3A2. J Immunother Cancer (2021) 9(6):2020–002140. doi: 10.1136/jitc-2020-002140 PMC8194339 34112739

[59] Saul L Josephs DH Cutler K Bradwell A Karagiannis P Selkirk C. Comparative Reactivity of Human IgE to Cynomolgus Monkey and Human Effector Cells and Effects on IgE Effector Cell Potency. MAbs (2014) 6(2):509–22. doi: 10.4161/mabs.27828 PMC3984339 24492303

[60] ClinicalTrials. Phase I Study of MOv18 IgE, a First in Class Chimeric IgE Antibody in Patients With Advanced Solid Tumours. Available at: https://clinicaltrialsgov/ct2/show/NCT02546921 (Accessed may 7, 2021).

[61] Fazekas J Furdos I Singer J Jensen-Jarolim E. Why Man’s Best Friend, the Dog, Could Also Benefit From an Anti-HER-2 Vaccine. Oncol Lett (2016) 12(4):2271–6. doi: 10.3892/ol.2016.5001 PMC5038860 27698788

[62] Grujic M Paivandy A Gustafson AM Thomsen AR Ohrvik H Pejler G. The Combined Action of Mast Cell Chymase, Tryptase and Carboxypeptidase A3 Protects Against Melanoma Colonization of the Lung. Oncotarget (2017) 8(15):25066–79. doi: 10.18632/oncotarget.15339 PMC5421910 28212574

[63] Williams IP Crescioli S Sow HS Bax HJ Hobbs C Ilieva KM. *In Vivo* Safety Profile of a CSPG4-Directed IgE Antibody in an Immunocompetent Rat Model. MAbs (2020) 12(1):1685349. doi: 10.1080/19420862.2019.1685349 31769737 PMC6927758

[64] Spicer J Basu B Montes A Banerji U Kristeleit R Veal GJ. Abstract CT141: Phase 1 Trial of MOv18, A First-in-Class IgE Antibody Therapy for Cancer. Cancer Res (2020) 80(16 Supplement):CT141. doi: 10.1158/1538-7445.AM2020-CT141

[65] Nakamura M Souri EA Osborn G Laddach R Chauhan J Stavraka C. IgE Activates Monocytes From Cancer Patients to Acquire a Pro-Inflammatory Phenotype. Cancers (Basel) (2020) 12(11):3376. doi: 10.3390/cancers12113376 PMC7698027 33203088

[66] Pellizzari G Hoskin C Crescioli S Mele S Gotovina J Chiaruttini G. IgE Re-Programs Alternatively-Activated Human Macrophages Towards Pro-Inflammatory Anti-Tumoural States. EBioMedicine (2019) 43:67–81. doi: 10.1016/j.ebiom.2019.03.080 30956175 PMC6562024

[67] Varricchi G Galdiero MR Loffredo S Marone G Iannone R Marone G. Are Mast Cells MASTers in Cancer? Front Immunol (2017) 8:424. doi: 10.3389/fimmu.2017.00424 28446910 PMC5388770

[68] Chan JK Magistris A Loizzi V Lin F Rutgers J Osann K. Mast Cell Density, Angiogenesis, Blood Clotting, and Prognosis in Women With Advanced Ovarian Cancer. Gynecol Oncol (2005) 99(1):20–5. doi: 10.1016/j.ygyno.2005.05.042 16055178

[69] Fu H Zhu Y Wang Y Liu Z Zhang J Wang Z. Tumor Infiltrating Mast Cells (TIMs) Confers a Marked Survival Advantage in Nonmetastatic Clear-Cell Renal Cell Carcinoma. Ann Surg Oncol (2017) 24(5):1435–42. doi: 10.1245/s10434-016-5702-5 27896514

[70] Hedstrom G Berglund M Molin D Fischer M Nilsson G Thunberg U. Mast Cell Infiltration Is a Favourable Prognostic Factor in Diffuse Large B-Cell Lymphoma. Br J Haematol (2007) 138(1):68–71. doi: 10.1111/j.1365-2141.2007.06612.x 17555448

[71] Yang XD Ai W Asfaha S Bhagat G Friedman RA Jin G. Histamine Deficiency Promotes Inflammation-Associated Carcinogenesis Through Reduced Myeloid Maturation and Accumulation of CD11b+Ly6G+ Immature Myeloid Cells. Nat Med (2011) 17(1):87–95. doi: 10.1038/nm.2278 21170045 PMC3075560

[72] Siebenhaar F Metz M Maurer M. Mast Cells Protect From Skin Tumor Development and Limit Tumor Growth During Cutaneous *De Novo* Carcinogenesis in a Kit-Dependent Mouse Model. Exp Dermatol (2014) 23(3):159–64. doi: 10.1111/exd.12328 24444017

[73] Cherdantseva TM Bobrov IP Avdalyan AM Klimachev VV Kazartsev AV Kryuchkova NG. Mast Cells in Renal Cancer: Clinical Morphological Correlations and Prognosis. Bull Exp Biol Med (2017) 163(6):801–4. doi: 10.1007/s10517-017-3907-7 29063337

[74] Dantas RCM de Souza RO Valverde LF Vidal MTA Sales CBS Sousa LP. Evaluation of Mast Cell Density in the Tumor Microenvironment in Oral Epithelial Dysplasia and Oral Squamous Cell Carcinoma. Appl Immunohistochem Mol Morphol (2017) 25(10):e83–8. doi: 10.1097/PAI.0000000000000587 29116959

[75] Brockmeyer P Kling A Schulz X Perske C Schliephake H Hemmerlein B. High Mast Cell Density Indicates a Longer Overall Survival in Oral Squamous Cell Carcinoma. Sci Rep (2017) 7(1):14677. doi: 10.1038/s41598-017-15406-5 29116177 PMC5677084

[76] Welsh TJ Green RH Richardson D Waller DA O’Byrne KJ Bradding P. Macrophage and Mast-Cell Invasion of Tumor Cell Islets Confers a Marked Survival Advantage in Non-Small-Cell Lung Cancer. J Clin Oncol (2005) 23(35):8959–67. doi: 10.1200/JCO.2005.01.4910 16219934

[77] Shikotra A Ohri CM Green RH Waller DA Bradding P. Mast Cell Phenotype, TNFalpha Expression and Degranulation Status in Non-Small Cell Lung Cancer. Sci Rep (2016) 6:38352. doi: 10.1038/srep38352 27922077 PMC5138591

[78] Sinnamon MJ Carter KJ Sims LP Lafleur B Fingleton B Matrisian LM. A Protective Role of Mast Cells in Intestinal Tumorigenesis. Carcinogenesis (2008) 29(4):880–6. doi: 10.1093/carcin/bgn040 PMC9640162 18258601

[79] Carlini MJ Dalurzo MC Lastiri JM Smith DE Vasallo BC Puricelli LI. Mast Cell Phenotypes and Microvessels in Non-Small Cell Lung Cancer and Its Prognostic Significance. Hum Pathol (2010) 41(5):697–705. doi: 10.1016/j.humpath.2009.04.029 20040391

[80] Oldford SA Haidl ID Howatt MA Leiva CA Johnston B Marshall JS. A Critical Role for Mast Cells and Mast Cell-Derived IL-6 in TLR2-Mediated Inhibition of Tumor Growth. J Immunol (2010) 185(11):7067–76. doi: 10.4049/jimmunol.1001137 21041732

[81] Purwar R Schlapbach C Xiao S Kang HS Elyaman W Jiang X. Robust Tumor Immunity to Melanoma Mediated by Interleukin-9-Producing T Cells. Nat Med (2012) 18(8):1248–53. doi: 10.1038/nm.2856 PMC3518666 22772464

[82] Rabelo Melo F Santosh Martin S Sommerhoff CP Pejler G. Exosome-Mediated Uptake of Mast Cell Tryptase Into the Nucleus of Melanoma Cells: A Novel Axis for Regulating Tumor Cell Proliferation and Gene Expression. Cell Death Dis (2019) 10(9):659. doi: 10.1038/s41419-019-1879-4 31506436 PMC6736983

[83] Fleischmann A Schlomm T Kollermann J Sekulic N Huland H Mirlacher M. Immunological Microenvironment in Prostate Cancer: High Mast Cell Densities Are Associated With Favorable Tumor Characteristics and Good Prognosis. Prostate (2009) 69(9):976–81. doi: 10.1002/pros.20948 19274666

[84] Foroozan M Roudi R Abolhasani M Gheytanchi E Mehrazma M. Clinical Significance of Endothelial Cell Marker CD34 and Mast Cell Marker CD117 in Prostate Adenocarcinoma. Pathol Res Pract (2017) 213(6):612–8. doi: 10.1016/j.prp.2017.04.027 28552539

[85] Hempel HA Cuka NS Kulac I Barber JR Cornish TC Platz EA. Low Intratumoral Mast Cells Are Associated With a Higher Risk of Prostate Cancer Recurrence. Prostate (2017) 77(4):412–24. doi: 10.1002/pros.23280 27868214

[86] Tan SY Fan Y Luo HS Shen ZX Guo Y Zhao LJ. Prognostic Significance of Cell Infiltrations of Immunosurveillance in Colorectal Cancer. World J Gastroenterol (2005) 11(8):1210–4. doi: 10.3748/wjg.v11.i8.1210 PMC4250716 15754407

[87] Naik R Baliga P Bansal R Pai M. Distribution of Mast Cells in the Axillary Lymph Nodes of Breast Cancer Patients. J Indian Med Assoc (1997) 95(12):606–7. 9586403

[88] Dabiri S Huntsman D Makretsov N Cheang M Gilks B Bajdik C. The Presence of Stromal Mast Cells Identifies a Subset of Invasive Breast Cancers With a Favorable Prognosis. Mod Pathol (2004) 17(6):690–5. doi: 10.1038/modpathol.3800094 15044916

[89] Ribatti D Finato N Crivellato E Guidolin D Longo V Mangieri D. Angiogenesis and Mast Cells in Human Breast Cancer Sentinel Lymph Nodes With and Without Micrometastases. Histopathology (2007) 51(6):837–42. doi: 10.1111/j.1365-2559.2007.02869.x 17944928

[90] Rajput AB Turbin DA Cheang MC Voduc DK Leung S Gelmon KA. Stromal Mast Cells in Invasive Breast Cancer Are a Marker of Favourable Prognosis: A Study of 4,444 Cases. Breast Cancer Res Treat (2008) 107(2):249–57. doi: 10.1007/s10549-007-9546-3 PMC2137942 17431762

[91] Amini RM Aaltonen K Nevanlinna H Carvalho R Salonen L Heikkila P. Mast Cells and Eosinophils in Invasive Breast Carcinoma. BMC Cancer (2007) 7:165. doi: 10.1186/1471-2407-7-165 17727696 PMC2048965

[92] della Rovere F Granata A Familiari D D’Arrigo G Mondello B Basile G. Mast Cells in Invasive Ductal Breast Cancer: Different Behavior in High and Minimum Hormone-Receptive Cancers. Anticancer Res (2007) 27(4B):2465–71. 17695540

[93] Mercogliano MF Bruni S Mauro F Elizalde PV Schillaci R. Harnessing Tumor Necrosis Factor Alpha to Achieve Effective Cancer Immunotherapy. Cancers (Basel) (2021) 13(3):564. doi: 10.3390/cancers13030564 33540543 PMC7985780

[94] Nicoud MB Sterle HA Massari NA Taquez Delgado MA Formoso K Herrero Ducloux MV. Study of the Antitumour Effects and the Modulation of Immune Response by Histamine in Breast Cancer. Br J Cancer (2020) 122(3):348–60. doi: 10.1038/s41416-019-0636-x PMC7000401 31748740

[95] Yan WL Shen KY Tien CY Chen YA Liu SJ. Recent Progress in GM-CSF-Based Cancer Immunotherapy. Immunotherapy (2017) 9(4):347–60. doi: 10.2217/imt-2016-0141 28303764

[96] Taskinen M Karjalainen-Lindsberg ML Leppa S. Prognostic Influence of Tumor-Infiltrating Mast Cells in Patients With Follicular Lymphoma Treated With Rituximab and CHOP. Blood (2008) 111(9):4664–7. doi: 10.1182/blood-2007-11-125823 18309035

[97] Molin D Edstrom A Glimelius I Glimelius B Nilsson G Sundstrom C. Mast Cell Infiltration Correlates With Poor Prognosis in Hodgkin’s Lymphoma. Br J Haematol (2002) 119(1):122–4. doi: 10.1046/j.1365-2141.2002.03768.x 12358914

[98] Englund A Molin D Enblad G Karlen J Glimelius I Ljungman G. The Role of Tumour-Infiltrating Eosinophils, Mast Cells and Macrophages in Classical and Nodular Lymphocyte Predominant Hodgkin Lymphoma in Children. Eur J Haematol (2016) 97(5):430–8. doi: 10.1111/ejh.12747 26872637

[99] Andersen MD Kamper P Nielsen PS Bendix K Riber-Hansen R Steiniche T. Tumour-Associated Mast Cells in Classical Hodgkin’s Lymphoma: Correlation With Histological Subtype, Other Tumour-Infiltrating Inflammatory Cell Subsets and Outcome. Eur J Haematol (2016) 96(3):252–9. doi: 10.1111/ejh.12583 25963595

[100] Banat GA Tretyn A Pullamsetti SS Wilhelm J Weigert A Olesch C. Immune and Inflammatory Cell Composition of Human Lung Cancer Stroma. PLoS One (2015) 10(9):e0139073. doi: 10.1371/journal.pone.0139073 26413839 PMC4587668

[101] Giannou AD Marazioti A Spella M Kanellakis NI Apostolopoulou H Psallidas I. Mast Cells Mediate Malignant Pleural Effusion Formation. J Clin Invest (2015) 125(6):2317–34. doi: 10.1172/JCI79840 PMC4497757 25915587

[102] Holzel M Landsberg J Glodde N Bald T Rogava M Riesenberg S. A Preclinical Model of Malignant Peripheral Nerve Sheath Tumor-Like Melanoma Is Characterized by Infiltrating Mast Cells. Cancer Res (2016) 76(2):251–63. doi: 10.1158/0008-5472.CAN-15-1090 26511633

[103] Maltby S Khazaie K McNagny KM. Mast Cells in Tumor Growth: Angiogenesis, Tissue Remodelling and Immune-Modulation. Biochim Biophys Acta (2009) 1796(1):19–26. doi: 10.1016/j.bbcan.2009.02.001 19233249 PMC2731828

[104] Ribatti D Crivellato E. Mast Cells, Angiogenesis, and Tumour Growth. Biochim Biophys Acta (2012) 1822(1):2–8. doi: 10.1016/j.bbadis.2010.11.010 21130163

[105] Komi DEA Redegeld FA. Role of Mast Cells in Shaping the Tumor Microenvironment. Clin Rev Allergy Immunol (2020) 58(3):313–25. doi: 10.1007/s12016-019-08753-w PMC7244463 31256327

[106] Ranieri G Ammendola M Patruno R Celano G Zito FA Montemurro S. Tryptase-Positive Mast Cells Correlate With Angiogenesis in Early Breast Cancer Patients. Int J Oncol (2009) 35(1):115–20. doi: 10.3892/ijo_00000319 19513558

[107] Aponte-Lopez A Fuentes-Panana EM Cortes-Munoz D Munoz-Cruz S. Mast Cell, the Neglected Member of the Tumor Microenvironment: Role in Breast Cancer. J Immunol Res (2018) 2018:2584243. doi: 10.1155/2018/2584243 29651440 PMC5832101

[108] Ammendola M Sacco R Sammarco G Luposella M Patruno R Gadaleta CD. Mast Cell-Targeted Strategies in Cancer Therapy. Transfus Med Hemother (2016) 43(2):109–13. doi: 10.1159/000444942 PMC4909357 27330532

[109] Rigoni A Colombo MP Pucillo C. The Role of Mast Cells in Molding the Tumor Microenvironment. Cancer Microenviron (2015) 8(3):167–76. doi: 10.1007/s12307-014-0152-8 PMC4715001 25194694

[110] Piliponsky AM Chen C-C Grimbaldeston MA Burns-Guydish SM Hardy J Kalesnikoff J. Mast Cell-Derived TNF can Exacerbate Mortality During Severe Bacterial Infections in C57BL/6-KitW-Sh/W-Sh Mice. Am J Pathol (2010) 176(2):926–38. doi: 10.2353/ajpath.2010.090342 PMC2808097 20035049

[111] Rodewald HR Feyerabend TB. Widespread Immunological Functions of Mast Cells: Fact or Fiction? Immunity (2012) 37(1):13–24. doi: 10.1016/j.immuni.2012.07.007 22840840

[112] Katz HR Austen KF. Mast Cell Deficiency, a Game of Kit and Mouse. Immunity (2011) 35(5):668–70. doi: 10.1016/j.immuni.2011.11.004 22118523

[113] Ribatti D. Mast Cells in Lymphomas. Crit Rev Oncol Hematol (2016) 101:207–12. doi: 10.1016/j.critrevonc.2016.03.016 27033307

[114] Aaltomaa S Lipponen P Papinaho S Kosma VM. Mast Cells in Breast Cancer. Anticancer Res (1993) 13:785–8. 8317912

[115] Mestas J Hughes CC. Of Mice and Not Men: Differences Between Mouse and Human Immunology. J Immunol (2004) 172(5):2731–8. doi: 10.4049/jimmunol.172.5.2731 14978070

[116] Passante E. Mast Cell and Basophil Cell Lines: A Compendium. Methods Mol Biol (2014) 1192:101–13. doi: 10.1007/978-1-4939-1173-8_8 25149487

[117] Epstein MM. Do Mouse Models of Allergic Asthma Mimic Clinical Disease? Int Arch Allergy Immunol (2004) 133(1):84–100. doi: 10.1159/000076131 14726635

[118] Finkelman FD. Anaphylaxis: Lessons From Mouse Models. JAllergy Clin Immunol (2007) 120(3):506–15. doi: 10.1016/j.jaci.2007.07.033 17765751

[119] Maurer M Taube C Schroder NWJ Ebmeyer J Siebenhaar F Geldmacher A. Mast Cells Drive IgE-Mediated Disease But may be Bystanders in Many Other Inflammatory and Neoplastic Conditions. J Allergy Clin Immunol (2019) 144(4):S19–30. doi: 10.1016/j.jaci.2019.07.017 31369803

[120] Bischoff SC. Role of Mast Cells in Allergic and Non-Allergic Immune Responses: Comparison of Human and Murine Data. Nat Rev Immunol (2007) 7(2):93–104. doi: 10.1038/nri2018 17259966

[121] Zschaler J Schlorke D Arnhold J. Differences in Innate Immune Response Between Man and Mouse. Crit Rev Immunol (2014) 34(5):433–54. doi: 10.1615/CritRevImmunol.2014011600 25404048

[122] Galli SJ Tsai M Piliponsky AM. The Development of Allergic Inflammation. Nature (2008) 454(7203):445–54. doi: 10.1038/nature07204 PMC3573758 18650915

[123] Akula S Paivandy A Fu Z Thorpe M Pejler G Hellman L. How Relevant Are Bone Marrow-Derived Mast Cells (BMMCs) as Models for Tissue Mast Cells? A Comparative Transcriptome Analysis of BMMCs and Peritoneal Mast Cells. Cells (2020) 9(9):2118. doi: 10.3390/cells9092118 PMC7564378 32957735

[124] Zhao W Kepley CL Morel PA Okumoto LM Fukuoka Y Schwartz LB. Fc Gamma RIIa, Not Fc Gamma RIIb, Is Constitutively and Functionally Expressed on Skin-Derived Human Mast Cells. J Immunol (2006) 177(1):694–701. doi: 10.4049/jimmunol.177.1.694 16785568 PMC2176083

[125] Yu Y Blokhuis BR Garssen J Redegeld FA. Non-IgE Mediated Mast Cell Activation. Eur J Pharmacol (2016) 778:33–43. doi: 10.1016/j.ejphar.2015.07.017 26164792

[126] Bruhns P. Properties of Mouse and Human IgG Receptors and Their Contribution to Disease Models. Blood (2012) 119(24):5640–9. doi: 10.1182/blood-2012-01-380121 22535666

[127] Xing W Austen KF Gurish MF Jones TG. Protease Phenotype of Constitutive Connective Tissue and of Induced Mucosal Mast Cells in Mice Is Regulated by the Tissue. Proc Natl Acad Sci USA (2011) 108(34):14210–5. doi: 10.1073/pnas.1111048108 PMC3161524 21825171

[128] Dai H Korthuis RJ. Mast Cell Proteases and Inflammation. Drug Discovery Today Dis Models (2011) 8(1):47–55. doi: 10.1016/j.ddmod.2011.06.004 PMC3223931 22125569

[129] Chauhan J McCraw AJ Nakamura M Osborn G Sow HS Cox VF. IgE Antibodies Against Cancer: Efficacy and Safety. Antibodies (2020) 9(4):55. doi: 10.3390/antib9040055 PMC7709114 33081206

[130] Pellizzari G Bax HJ Josephs DH Gotovina J Jensen-Jarolim E Spicer JF. Harnessing Therapeutic IgE Antibodies to Re-Educate Macrophages Against Cancer. Trends Mol Med (2020) 26(6):615–26. doi: 10.1016/j.molmed.2020.03.002 32470387

[131] Taniguchi S Elhance A Van Duzer A Kumar S Leitenberger JJ Oshimori N. Tumor-Initiating Cells Establish an IL-33–TGF-β Niche Signaling Loop to Promote Cancer Progression. Science (2020) 369(6501):eaay1813. doi: 10.1126/science.aay1813 32675345 PMC10870826

[132] Kamphuis JBJ Worrall WPM Stackowicz J Mougel A Mauré E Conde E. Comment on “Tumor-Initiating Cells Establish an IL-33-TGF-β Niche Signaling Loop to Promote Cancer Progression”. Science (2021) 372(6538):eabf2022. doi: 10.1126/science.abf2022 33833094

[133] Duan Z Luo Y. Targeting Macrophages in Cancer Immunotherapy. Signal Transduct Target Ther (2021) 6(1):127. doi: 10.1038/s41392-021-00506-6 33767177 PMC7994399

[134] Cotechini T Atallah A Grossman A. Tissue-Resident and Recruited Macrophages in Primary Tumor and Metastatic Microenvironments: Potential Targets in Cancer Therapy. Cells (2021) 10(4):960. doi: 10.3390/cells10040960 33924237 PMC8074766

[135] Mehraj U Qayoom H Mir MA. Prognostic Significance and Targeting Tumor-Associated Macrophages in Cancer: New Insights and Future Perspectives. Breast Cancer (2021) 28(3):539–55. doi: 10.1007/s12282-021-01231-2 33661479

[136] Sihra BS Kon OM Grant JA Kay AB. Expression of High-Affinity IgE Receptors (FcϵRI) on Peripheral Blood Basophils, Monocytes, and Eosinophils in Atopic and Nonatopic Subjects: Relationship to Total Serum IgE Concentrations. J Allergy Clin Immunol (1997) 99(5):699–706. doi: 10.1016/S0091-6749(97)70033-2 9155838

[137] Maurer D Fiebiger E Reininger B Wolff-Winiski B Jouvin MH Kilgus O. Expression of Functional High Affinity Immunoglobulin E Receptors (Fc Epsilon RI) on Monocytes of Atopic Individuals. J Exp Med (1994) 179(2):745–50. doi: 10.1084/jem.179.2.745 PMC2191351 8294882

[138] Ginhoux F Jung S. Monocytes and Macrophages: Developmental Pathways and Tissue Homeostasis. Nat Rev Immunol (2014) 14(6):392–404. doi: 10.1038/nri3671 24854589

[139] Bannert C Bidmon-Fliegenschnee B Stary G Hotzy F Stift J Nurko S. Fc-Epsilon-RI, the High Affinity IgE-Receptor, Is Robustly Expressed in the Upper Gastrointestinal Tract and Modulated by Mucosal Inflammation. PLoS One (2012) 7(7):e42066. doi: 10.1371/journal.pone.0042066 22848703 PMC3407106

[140] MacDermot J Joseph S Dollery CT. The Identity of IgE Receptors (Fc Epsilon RII) That Mediate Cellular Activation of Human Macrophages: Evidence Against a Role for CD23. Mol Immunol (1992) 29(1):71–6. doi: 10.1016/0161-5890(92)90158-T 1530985

[141] Osterhoff B Rappersberger K Wang B Koszik F Ochiai K Kinet JP. Immunomorphologic Characterization of Fc Epsilon RI-Bearing Cells Within the Human Dermis. J Invest Dermatol (1994) 102(3):315–20. doi: 10.1111/1523-1747.ep12371789 8120415

[142] Escribese MM Rosace D Chivato T Fernandez TD Corbi AL Barber D. Alternative Anaphylactic Routes: The Potential Role of Macrophages. Front Immunol (2017) 8:515. doi: 10.3389/fimmu.2017.00515 28533777 PMC5421149

[143] Finkelman FD Khodoun MV Strait R. Human IgE-Independent Systemic Anaphylaxis. J Allergy Clin Immunol (2016) 137(6):1674–80. doi: 10.1016/j.jaci.2016.02.015 PMC7607869 27130857

[144] Joseph M Tonnel AB Capron A Dessaint JP. The Interaction of IgE Antibody With Human Alveolar Macrophages and Its Participation in the Inflammatory Processes of Lung Allergy. Agents Actions (1981) 11(6-7):619–22. doi: 10.1007/BF01978766 7340451

[145] Joseph M Tonnel AB Torpier G Capron A Arnoux B Benveniste J. Involvement of Immunoglobulin E in the Secretory Processes of Alveolar Macrophages From Asthmatic Patients. J Clin Invest (1983) 71(2):221–30. doi: 10.1172/JCI110762 PMC436860 6185540

[146] Capron M Jouault T Prin L Joseph M Ameisen JC Butterworth AE. Functional Study of a Monoclonal Antibody to IgE Fc Receptor (Fc Epsilon R2) of Eosinophils, Platelets, and Macrophages. J Exp Med (1986) 164(1):72–89. doi: 10.1084/jem.164.1.72 2425032 PMC2188189

[147] Melewicz FM Plummer JM Spiegelberg HL. Comparison of the Fc Receptors for IgE on Human Lymphocytes and Monocytes. J Immunol (1982) 129(2):563–9. 6211489

[148] Spiegelberg HL Boltz-Nitulescu G Plummer JM Melewicz FM. Characterization of the IgE Fc Receptors on Monocytes and Macrophages. Fed Proc (1983) 42(1):124–8. 6401249

[149] Noro N Yoshioka A Adachi M Yasuda K Masuda T Yodoi J. Monoclonal Antibody (H107) Inhibiting IgE Binding to Fc Epsilon R(+) Human Lymphocytes. J Immunol (1986) 137(4):1258–63. 2942602

[150] Kinet JP. The High-Affinity IgE Receptor (Fc Epsilon RI): From Physiology to Pathology. AnnuRevImmunol (1999) 17:931–72. doi: 10.1146/annurev.immunol.17.1.931 10358778

[151] Irani AM Huang C Xia HZ Kepley C Nafie A Fouda ED. Immunohistochemical Detection of Human Basophils in Late-Phase Skin Reactions. J Allergy Clin Immunol (1998) 101(3):354–62. doi: 10.1016/S0091-6749(98)70248-9 9525452

[152] Kita H Gleich GJ. Eosinophils and IgE Receptors: A Continuing Controversy. Blood (1997) 89(10):3497–501. doi: 10.1182/blood.V89.10.3497 9160653

[153] Ishizuka T Okayama Y Kobayashi H Mori M. Interleukin-10 Is Localized to and Released by Human Lung Mast Cells. Clin Exp Allergy (1999) 29(10):1424–32. doi: 10.1046/j.1365-2222.1999.00636.x 10520066

[154] Baghestanian M Hofbauer R Kiener HP Bankl HC Wimazal F Willheim M. The C-Kit Ligand Stem Cell Factor and Anti-IgE Promote Expression of Monocyte Chemoattractant Protein-1 in Human Lung Mast Cells. Blood (1997) 90(11):4438–49. doi: 10.1182/blood.V90.11.4438.4438_4438_4449 9373254

[155] da Silva EZ Jamur MC Oliver C. Mast Cell Function: A New Vision of an Old Cell. J Histochem Cytochem (2014) 62(10):698–738. doi: 10.1369/0022155414545334 25062998 PMC4230976

[156] Casale TB Wood D Richerson HB Zehr B Zavala D Hunninghake GW. Direct Evidence of a Role for Mast Cells in the Pathogenesis of Antigen-Induced Bronchoconstriction. J Clin Invest (1987) 80(5):1507–11. doi: 10.1172/JCI113234 PMC442412 3680512

[157] Maciel TT Moura IC Hermine O. The Role of Mast Cells in Cancers. F1000Prime Rep (2015) 7:09. doi: 10.12703/P7-09 25705392 PMC4311277

[158] Schleimer RP MacGlashan DW Jr. Schulman ES Peters SP Adams GK Adkinson NF Jr.. Human Mast Cells and Basophils–Structure, Function, Pharmacology, and Biochemistry. ClinRevAllergy (1983) 1:327–41. doi: 10.1007/BF02991224 6201252

[159] Turner H Kinet JP. Signalling Through the High-Affinity IgE Receptor Fc epsilonRI. Nature (1999) 402(6760 Suppl):B24–30. doi: 10.1038/35037021 10586892

[160] Saxena S Singh A Singh P. Tumor Associated Mast Cells: Biological Roles and Therapeutic Applications. Anat Cell Biol (2020) 53(3):245–51. doi: 10.5115/acb.19.181 PMC7527126 32879056

[161] Gould HJ Sutton BJ. IgE in Allergy and Asthma Today. Nat Rev Immunol (2008) 8(3):205–17. doi: 10.1038/nri2273 18301424

[162] Folkerts J Gaudenzio N Maurer M Hendriks RW Stadhouders R Tam SY. Rapid Identification of Human Mast Cell Degranulation Regulators Using Functional Genomics Coupled to High-Resolution Confocal Microscopy. Nat Protoc (2020) 15(3):1285–310. doi: 10.1038/s41596-019-0288-6 PMC7197894 32060492

[163] Dela Cruz JS Trinh KR Morrison SL Penichet ML. Recombinant Anti-Human HER2/neu IgG3-(GM-CSF) Fusion Protein Retains Antigen Specificity and Cytokine Function and Demonstrates Antitumor Activity. J Immunol (2000) 165(9):5112–21. doi: 10.4049/jimmunol.165.9.5112 11046042

[164] Shiau MY Chiou HL Lee YL Kuo TM. & Chang, Y. H.: Establishment of a Consistent L929 Bioassay System for TNF-α Quantitation to Evaluate the Effect of Lipopolysaccharide, Phytomitogens and Cytodifferentiation Agents on Cytotoxicity of TNF-α Secreted by Adherent Human Mononuclear Cells. Mediators Inflamm (2001) 10(4):199–208. doi: 10.1080/09629350123139 11577996 PMC1781708

[165] Zanata F Shaik S Devireddy RV Wu X Ferreira LM Gimble JM. Cryopreserved Adipose Tissue-Derived Stromal/Stem Cells: Potential for Applications in Clinic and Therapy. Adv Exp Med Biol (2016) 951:137–46. doi: 10.1007/978-3-319-45457-3_11 27837560

[166] Lim MH Ong WK Sugii S. The Current Landscape of Adipose-Derived Stem Cells in Clinical Applications. Expert Rev Mol Med (2014) 16:e8. doi: 10.1017/erm.2014.8 24807467

[167] Conrad DH Wingard JR Ishizaka T. The Interaction of Human and Rodent IgE With the Human Basophil IgE Receptor. J Immunol (1983) 130(1):327–33. 6183355

[168] Achatz G. The Biology of IgE: Molecular Mechanism Restraining Potentially Dangerous High Serum IgE Titres *In Vivo* . In: Penichet M Jensen-Jarolim E. editors. Cancer and IgE. (2010). doi: 10.1007/978-1-60761-451-7_2

[169] Kubo S Nakayama T Matsuoka K Yonekawa H Karasuyama H. Long Term Maintenance of IgE-Mediated Memory in Mast Cells in the Absence of Detectable Serum IgE. J Immunol (2003) 170(2):775–80. doi: 10.4049/jimmunol.170.2.775 12517940

[170] Lawrence MG Woodfolk JA Schuyler AJ Stillman LC Chapman MD Platts-Mills TAE. Half-Life of IgE in Serum and Skin: Consequences for Anti-IgE Therapy in Patients With Allergic Disease. J Allergy Clin Immunol (2017) 139(2):422–8.e424. 27496596 10.1016/j.jaci.2016.04.056 PMC5405770

[171] Beck LA Marcotte GV MacGlashan D Togias A Saini S. Omalizumab-Induced Reductions in Mast Cell Fcϵri Expression and Function. J Allergy Clin Immunol (2004) 114(3):527–30. doi: 10.1016/j.jaci.2004.06.032 15356552

[172] Cass RM Andersen BR. The Disappearance Rate of Skin-Sensitizing Antibody Activity After Intradermal Administration. J Allergy (1968) 42(1):29–35. doi: 10.1016/0021-8707(68)90129-9 5243796

[173] Oettgen HC. Fifty Years Later: Emerging Functions of IgE Antibodies in Host Defense, Immune Regulation, and Allergic Diseases. J Allergy Clin Immunol (2016) 137(6):1631–45. doi: 10.1016/j.jaci.2016.04.009 PMC4898788 27263999

[174] Dellinger AL Cunin P Lee D Kung AL Brooks DB Zhou Z. Inhibition of Inflammatory Arthritis Using Fullerene Nanomaterials. PLoS One (2015) 10(4):e0126290. doi: 10.1371/journal.pone.0126290 25879437 PMC4400016

[175] Norton SK Dellinger A Zhou Z Lenk R Macfarland D Vonakis B. A New Class of Human Mast Cell and Peripheral Blood Basophil Stabilizers That Differentially Control Allergic Mediator Release. Clin Trans Sci (2010) 3(4):158–69. doi: 10.1111/j.1752-8062.2010.00212.x PMC5350695 20718816

[176] de Vries VC Wasiuk A Bennett KA Benson MJ Elgueta R Waldschmidt TJ. Mast Cell Degranulation Breaks Peripheral Tolerance. Am J Transplant (2009) 9(10):2270–80. doi: 10.1111/j.1600-6143.2009.02755.x PMC3808998 19681828

[177] Brudno JN Kochenderfer JN. Toxicities of Chimeric Antigen Receptor T Cells: Recognition and Management. Blood (2016) 127(26):3321–30. doi: 10.1182/blood-2016-04-703751 PMC4929924 27207799

[178] Fesnak AD June CH Levine BL. Engineered T Cells: The Promise and Challenges of Cancer Immunotherapy. Nat Rev Cancer (2016) 16(9):566–81. doi: 10.1038/nrc.2016.97 PMC5543811 27550819

[179] Martin-Antonio B Sune G Perez-Amill L Castella M Urbano-Ispizua A. Natural Killer Cells: Angels and Devils for Immunotherapy. Int J Mol Sci (2017) 18(9):1868. doi: 10.3390/ijms18091868 PMC5618517 28850071

[180] Gold S. Premedication Use in Preventing Acute Infliximab Infusion Reactions in Patients With Inflammatory Bowel Disease: A Single Center Cohort Study. Inflamm Bowel Dis (2017) 23(10):1882–9. doi: 10.1097/MIB.0000000000001189 28837521

[181] Touma W. Risk Factors for and Pre-Medications to Prevent Cetuximab-Induced Infusion Reactions in Patients With Squamous Cell Carcinoma of the Head and Neck. Oral Oncol (2014) 50(9):895–900. doi: 10.1016/j.oraloncology.2014.06.017 25037161 PMC4130782

[182] Northcutt M Al-Subu A Bella B Elitsur Y. Safety of Infliximab in Children With IBD: The Experience of an Academic Center in WV. West Va Med J (2014) 110(3):26–9. 24984403

[183] Sorokin P. Mylotarg™ Approved for Patients With CD33+ Acute Myeloid Leukemia. Clin J Oncol Nurs (2000) 4(6):279–80. 11899326

[184] Jensen-Jarolim E Achatz G Turner MC Karagiannis S Legrand F Capron M. AllergoOncology: The Role of IgE-Mediated Allergy in Cancer. Allergy (2008) 63(10):1255–66. doi: 10.1111/j.1398-9995.2008.01768.x PMC2999743 18671772

[185] Almagro JC Daniels-Wells TR Perez-Tapia SM Penichet ML. Progress and Challenges in the Design and Clinical Development of Antibodies for Cancer Therapy. Front Immunol (2017) 8:1751. doi: 10.3389/fimmu.2017.01751 29379493 PMC5770808

[186] Rosenbaum L. Tragedy, Perseverance, and Chance - The Story of CAR-T Therapy. N Engl J Med (2017) 377(14):1313–5. doi: 10.1056/NEJMp1711886 28902570

[187] Siegler EL Wang P. Preclinical Models in Chimeric Antigen Receptor-Engineered T-Cell Therapy. Hum Gene Ther (2018) 29(5):534–46. doi: 10.1089/hum.2017.243 29390873

[188] Porter DL Levine BL Kalos M Bagg A June CH. Chimeric Antigen Receptor-Modified T Cells in Chronic Lymphoid Leukemia. N Engl J Med (2011) 365(8):725–33. doi: 10.1056/NEJMoa1103849 PMC3387277 21830940

[189] Shimabukuro-Vornhagen A. Cytokine Release Syndrome. J Immunother Cancer (2018) 6(1):56. doi: 10.1186/s40425-018-0343-9 29907163 PMC6003181

